# Transcriptome Analysis of Transiently Reversible Cell Vacuolization Caused by Excessive Serum Concentration in *Scophthalmus maximus*

**DOI:** 10.3390/biology13070545

**Published:** 2024-07-19

**Authors:** Yuting Song, Lijun Shao, Xiaoli Yu

**Affiliations:** 1 School of Public Health, Shandong Second Medical University, Weifang 261053, China; shaolijun2002@163.com; 2 College of Marine Life Sciences, Ocean University of China, Qingdao 266003, China; sandysyt0720@163.com; 3 Institute of Evolution & Marine Biodiversity, Ocean University of China, Qingdao 266003, China

**Keywords:** cell vacuolization, stress repair, transcriptome analysis

## Abstract

**Simple Summary:**

The phenomenon of cellular vacuolization is closely related to apoptosis. During the culture of marine fish cell lines, an interesting phenomenon was found that the addition of excessive concentrations of serum to the culture medium could lead to the vacuolization of marine fish cells. However, this vacuolization phenomenon will disappear as the number of cell passages increases. Transcriptome sequencing was used to reveal the relationship between this transiently reversible phenomenon of cellular vacuolization and two influential factors, serum concentration and trypsin digestion. This study provides new insights into the mechanisms of cellular vacuolization and serves as a reference for the culture of marine fish cell lines.

**Abstract:**

As an important research tool, cell lines play a vital role in life science research, medical research, and drug development. During the culture of the *Scophthalmus maximus* head kidney (TK) cell line, we found a phenomenon of cell vacuolization caused by excessive serum concentration. Moreover, the vacuolization of the cells gradually disappeared after passage by trypsin digestion. In clarifying the formation mechanism of this reversible cellular vacuolation, transcriptomics was utilized to explore the mechanism of cell vacuolization caused by excessive serum concentration. Transcriptome analysis indicated that excessive serum concentration could cause the up-regulated expression of PORCN and other genes to promote cell proliferation. Compared with cells whose vacuolization disappeared after trypsin digestion and passage, the expression of mitosis-related genes (BUB1, ttk, Mad2, Cdc20, CDK1, CCNB1), nuclear stability-related genes LMNB1 and tissue stress and repair-related genes HMMR in vacuolated cells caused by excessive serum concentration was significantly up-regulated. There is a regulatory system related to adaptation and stress repair in the cells, which can maintain cell stability to a certain extent. This study provides a theoretical basis for the stable culture of fish cell lines and the solution to the problem of cell vacuolation.

## 1. Introduction

Cell vacuolization is frequently observed in experiments involving drug treatment, changes in the acid-base balance, and viral infection [1,2,3]. In studies on mammalian cells, it has been found that vacuolization caused by exposure to inducers is transiently reversible, whereas irreversible vacuolization marks the end of cell death [4]. Irreversible vacuolization happens when vacuoles form in cells and cannot revert to their normal state, even after the removal of the stressor. This type of vacuolization is often associated with severe cellular damage and can lead to cell death. Experiments on the reversible vacuolization of mammalian cells induced by Henics et al. showed that no major metabolic damage was evident in the vacuolated cells [5]. Reversible vacuolization occurs when cells form vacuoles that can be reverted to their normal state if the stimulus causing the vacuolation is removed. This condition is typically not associated with cell death and can be a temporary response to various stressors. Cell vacuolization can be induced by conditions or spontaneously. In addition, cell vacuolization is also related to cell types, such as adherent cells, semi-adherent cells, and suspended cells [6]. Vacuoles in the cytoplasm can have multiple origins, such as the endoplasmic reticulum, endosomes, Golgi apparatus, and mitochondria [7]. Despite the abundance of data on vacuolization inducers, their formation mechanism remains unclear.

As an important research tool, fish cell lines have been widely used in research on virology, immunology, toxicology, genetics, developmental biology, and physiology. In-depth research on fish cell lines is of great significance both in theory and practical application. Turbot (*Scophthalmus maximus*) is a commercially cultured fish with high economic value, belonging to the family Bothidae [8]. The construction of turbot cell lines provides experimental materials for in vitro research on turbot virology, immunology and other basic biology. Currently reported turbot cell lines include the embryonic cell line, fin cell line, head kidney cell line and spleen cell line [9,10,11,12].

In the process of culturing the turbot head kidney (TK) cell line, we found a phenomenon of cell vacuolization caused by excessive serum concentration. After passaging through trypsin digestion, the vacuolization of the cells would disappear with the extension of the culture time. In addition, we also noticed that irreversible cellular vacuolization caused by excessive serum concentration or other conditions could lead to cell death. The results of the research on cell vacuolization at the end of the last century suggested that the formation of vacuolization is an adaptation to environmental changes and may lead to specific cell death [6]. However, studies in recent years tend to believe that vacuolization has no effect on cell death and may even reduce cell survival stress and increase the cell survival rate [4]. Currently, no studies have been conducted to determine whether this vacuolization phenomenon is beneficial for the growth of fish cell lines. In clarifying the formation mechanism of this cellular vacuolation, RNA-Seq technology was utilized to explore the relationship between cell vacuolization and the two influencing factors of serum concentration and trypsin digestion. This study provides a theoretical basis for the stable culture of fish cell lines and the solution to the problem of cell vacuolation.

## 2. Materials and Methods

### 2.1. Cell Culture and Treatment

TK cells were inoculated into 6-well plates at a density of 5 × 10^5^ cells per well. Upon 80–90% confluence, the original culture medium was replaced with 5% and 20%fetal bovine serum (FBS) (10,099,141, Gibco, Waltham, MA, USA), respectively [11]. The vacuolated cells were digested with trypsin (T1300, Solarbio, Beijing, China) and continued to be cultured until the vacuoles disappeared. To analyze the effect of serum concentration on cell vacuolization, TK cells cultured with 5% FBS medium were collected as a control group (TK5) and cells that became vacuolated after culture with 20% FBS medium were used as the experimental group (TK20). In another group, to study the effect of trypsin digestion on cell vacuolization, cells that recover from vacuolization to a normal state after passage were used as the experimental group (TKP). The Japanese flounder gills (FG) cell line was also treated in the same way to verify the ubiquity of this transiently reversible cellular vacuolization phenomenon in marine fish cell lines. The TK cell line was obtained from Dr. Zhihong Gong. The FG cell line was obtained from Dr. Zhan Gao [13].

### 2.2. cDNA Library Construction

The cells were harvested using Trizol reagent (Invitrogen, Carlsbad, CA, USA) to isolate the RNA. Take three replicate samples from each group. The RNA quality was assessed on an Agilent 2100 Bioanalyzer (Agilent Technologies, Palo Alto, CA, USA) and checked using RNase-free agarose gel electrophoresis. The mRNA was enriched by Oligo(dT) beads and reverse transcribed into cDNA using the NEB Next Ultra RNA Library Prep Kit for Illumina (NEB #7530, New England Biolabs, Ipswich, MA, USA).

### 2.3. RNA Sequencing

The cDNA library was sequenced using Illumina Novaseq6000 by Gene Denovo Biotechnology Co. (Guangzhou, China). Fastp (version 0.18.0) was used to filter out reads containing adapters, reads with an N ratio greater than 10% and reads that were all A bases. Reads with a base quality value Q ≤ 20 that account for more than 50% of the entire reads were also filtered out.

### 2.4. Bioinformatics Methods

HISAT2.2.4 was employed to map the paired-end clean reads to the reference genome of the turbot and StringTie v1.3.1 was then used to assemble these mapped reads into potential transcripts. RSEM was utilized to calculate the expression levels of the transcripts. The reproducibility of three replicate samples was assessed through correlation analysis. DESeq2 was used to perform differential expression analysis of the RNA-seq data. All differentially expressed genes (DEGs) were mapped to the Gene Ontology database and the Kyoto Encyclopedia of Genes and Genomes (KEGG) database by kobas software. Cytoscape (v3.9.1) software was used to visualize interaction networks and capture core genes.

### 2.5. Validation by qPCR Analysis

To verify the reliability of the transcriptome data, the expression of all core DEGs obtained through protein interaction network analysis was detected by quantitative PCR (qPCR). The cDNA was used as the template of qPCR. The primers for these core DEGs were designed by primer 5.0 software (Table S1). The relative gene expression levels were calculated by normalizing to the reference gene (*β-actin*).

## 3. Results

### 3.1. Effects of FBS Concentration and Trypsin Digestion on Cell Growth

**Figure 1:**
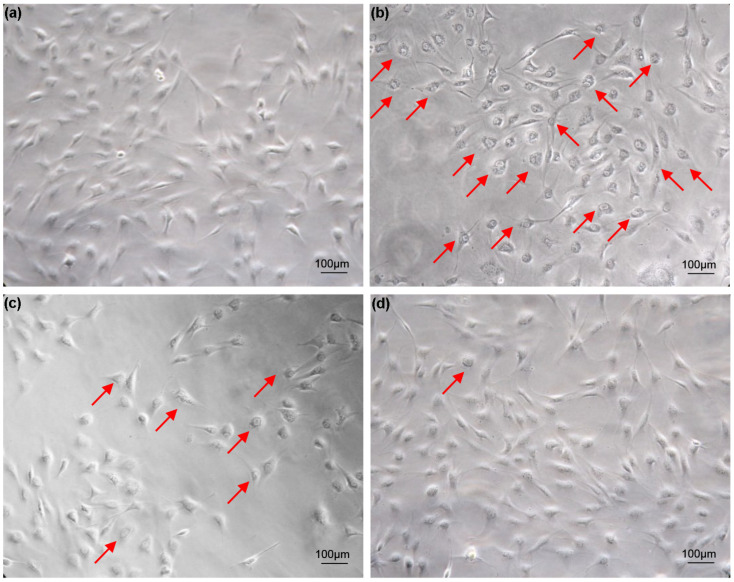
Vacuolization of turbot TK cell line caused by excessive serum concentration. (**a**) Cells cultured in 5% FBS medium for two weeks; (**b**) Cell vacuolation occurs after two weeks of culture in 20% FBS medium; (**c**) The vacuolization of cells improved after cells cultured in 20% FBS medium were digested and passaged with trypsin; (**d**) Cells cultured in 20% FBS medium were digested with trypsin and cultured until the cells return to normal from the vacuolated state. Arrows indicate vacuolated cells. Scale bars: 100 µm.

Cells grow well in 5% FBS medium, and vacuolization does not occur as the number of cell passages increases (Figure 1a). For cells cultured in 20% FBS medium, most cells appeared vacuolated after two weeks (Figure 1b). The vacuolated cells were trypsinized and inoculated into 6-well plates containing 20% FBS medium. As the number of passages increased, the vacuolation phenomenon of cells cultured in 20% FBS medium improved significantly (Figure 1c). After two months, the cell vacuolation phenomenon disappeared (Figure 1d). Cell vacuolization caused by excessive serum concentration is transiently reversible. This reversible cellular vacuolation induced by excessive concentrations of serum has also been verified in FG cell lines (Figure S1).

### 3.2. Transcriptome Sequencing and Identification of DEGs

**Figure 2:**
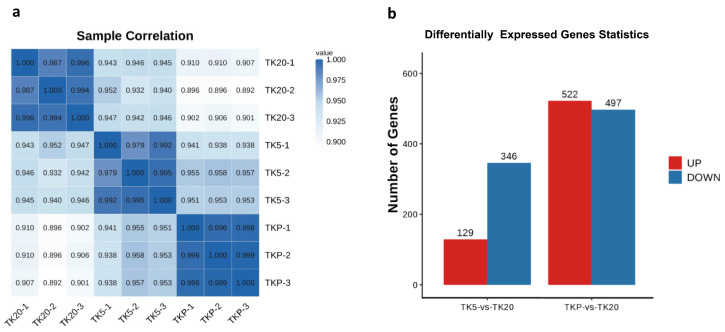
Pearson correlation coefficient analysis and identification of DEGs. (**a**) Pearson correlation coefficient between nine samples; (**b**) Identification of the number of DEGs in the TK5-TK20 group and TKP-TK20 group.

After removing low-quality reads, transcriptome sequencing yielded a total of 38,782,894–51,665,498 high-quality clean reads across all nine libraries. After filtering, the number of sequenced bases with a base quality value above Q20 accounts for more than 97% of raw reads, and the number of sequenced bases with a base quality value above Q30 accounts for 92% of raw reads. The GC content was ranged from 48.78 to 49.47%. The number of all reads that can be mapped to the reference genome and the proportion of effective reads reaches more than 70% (Table 1). The Pearson correlation coefficient of biological replicate samples within a group was significantly larger than the others and this demonstrates that there is a significant correlation between them. The large difference in Pearson correlation coefficients between the two groups proves that the grouping is meaningful. In this study, a total of 475 transcripts were identified as DEGs in the TK5-TK20 group and 1019 were identified as DEGs in the TKP-TK20 group (Figure 2b).

### 3.3. GO Enrichment Analysis

**Figure 3:**
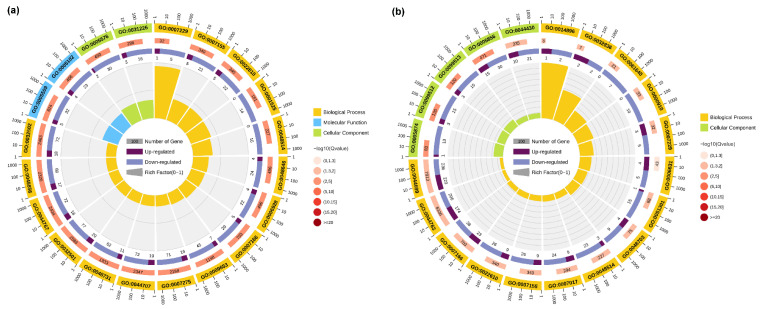
The GO analysis of DEGs in the TK5-TK20 group (**a**) and the TKP-TK20 group (**b**). The first circle: the top 20 enriched GO terms, and outside the circle is the coordinate scale of the number of DEGs; the second circle: the number of all transcripts and Q value in this GO term; the third circle: dark purple represents the proportion of up-regulated differential genes, and light purple represents the proportion of down-regulated differential genes; fourth circle: RichFactor value of each GO term (the number of DEGs divided by the total number of transcripts in the GO term).

To further investigate the biological processes and biological functions associated with the DEGs, GO analysis was performed. The top 20 GO terms enriched in the TK5-TK20 group and TK5-TKP group are listed in Table S2. The GO enrichment results indicated that the changes in the biological process of DEGs in the TK5-TK20 group were significantly enriched in the ‘integrin-mediated signaling pathway’. The changes in the molecular function of DEGs in TK5-TK20 group were enriched in the ‘calcium ion binding’ and ‘receptor binding’. The changes in the cellular component of DEGs in the TK5-TK20 group were enriched in the ‘extracellular region’ and ‘intrinsic component of the plasma membrane’ (Figure 3a). In the TKP-TK20 group, the changes in the biological process of DEGs were significantly enriched in the ‘muscle hypertrophy’ and ‘glomerular basement membrane development’. The changes in the cellular component of DEGs in the TKP-TK20 group were remarkably enriched in the ‘microtubule’, ‘supramolecular fiber’, and ‘polymeric cytoskeletal fiber’ (Figure 3b).

### 3.4. KEGG Enrichment Analysis

**Figure 4:**
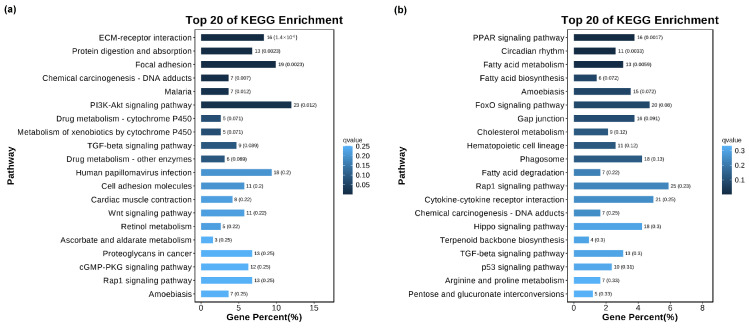
The KEGG analysis of DEGs in the TK5-TK20 group (**a**) and TKP-TK20 group (**b**).

The KEGG database was used to study signaling pathways related to cell vacuolization. KEGG enrichment analysis shows that the cell vacuolation-related DEGs in the TK5-TK20 group are significantly enriched in the ‘ECM-receptor interaction’, ‘Protein digestion and absorption’, ‘Focal adhesion’, ‘PI3K-Akt signaling pathway’, ‘Wnt signaling pathway’ et al. (Figure 4a). The DEGs in TK5-TKP are mainly enriched in the ‘PPAR signaling pathway’, ‘Circadian rhythm’, ‘Fatty acid metabolism’, ‘Amoebiasis’, ‘FoxO signaling pathway’, ‘Gap junction’, ‘Hematopoietic cell lineage’ and ‘Phagosome’ et al. (Figure 4b).

### 3.5. Protein–Protein Interaction (PPI) Analysis of DEGs Related to Cell Vacuolation

**Figure 5:**
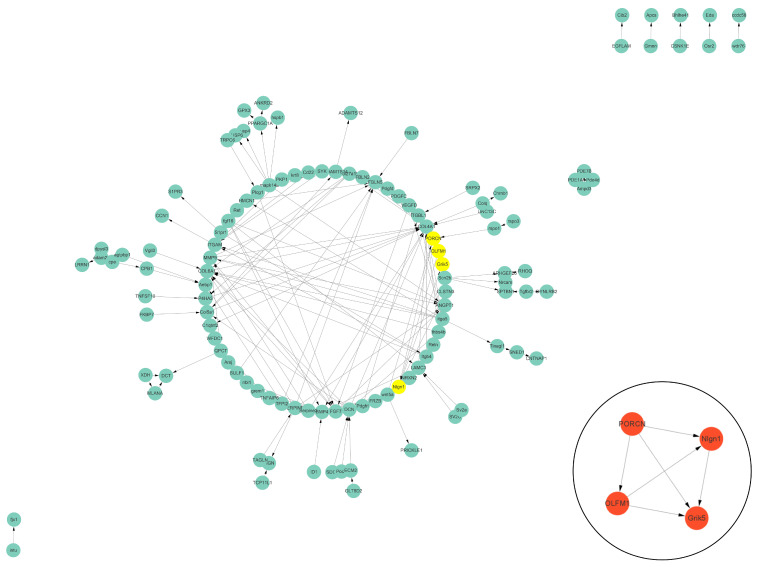
The PPI network of the TK5-TK20 group. Green nodes represent cell vacuolation-related differentially expressed proteins. Red and yellow nodes represent core cell vacuolation-related differentially expressed proteins. Arrows represent the direction of protein interaction.

**Figure 6:**
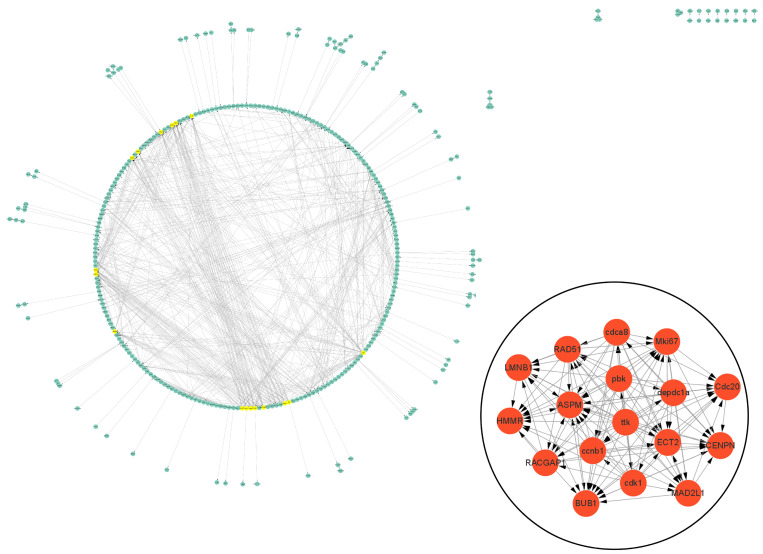
The PPI network of the TKP-TK20 group. Green nodes represent cell vacuolation-related differentially expressed proteins. Red and yellow nodes represent core cell vacuolation-related differentially expressed proteins. Arrows represent the direction of protein interaction.

The PPI data of cell vacuolation-related differential expressed proteins obtained from the string database was used to construct a visual network. The protein–protein interaction network of the TK5-TK20 group includes 120 nodes and 186 edges. The core cell vacuolation-related differential expressed proteins interaction network captured based on MCODE analysis includes four nodes and six edges (Figure 5). The protein–protein interaction network of the TK5-TKP group includes 343 nodes and 872 edges. The core cell vacuolation-related differential expressed proteins interaction network has 17 nodes and 121 edges (Figure 6). We screened out four DEGs in the TK5-TK20 group, including PORCN (porcupine O-acyltransferase), OLFM1 (Olfactomedin 1), Nlgn1 (neuroligin 1), and Grik5 (glutamate receptor, ionotropic, kainate 5). In the TK5-TKP group, LMNB1 (lamin B1), RAD51 (RAD51 recombinase), HMMR (hyaluronan-mediated motility receptor), RACGAP1 (Rac GTPase Activating Protein 1), BUB1 (BUB1 mitotic checkpoint serine/threonine kinase), MAD2L1 (MAD2 mitotic arrest deficient-like 1), CENPN (centromere protein N), Cdc20 (cell division cycle 20), Mki67 (marker of proliferation Ki-67), cdca8 (cell division cycle associated 8), ASPM (abnormal spindle microtubule assembly), ccnb1 (cyclin B1), cdk1 (cyclin-dependent kinase 1), ECT2 (epithelial cell transforming 2), depdc1a (DEP domain containing 1a), pbk, (PDZ binding kinase), and ttk (ttk protein kinase) were selected as core genes.

### 3.6. Bioinformatics Analysis of Interesting DEGs

**Figure 7:**
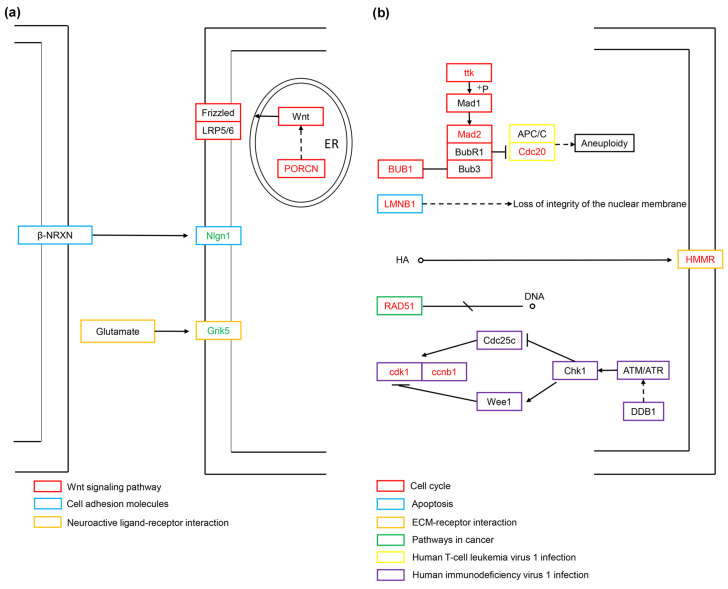
The regulatory mechanisms related to cell vacuolization for the TK5-TK20 group (**a**) and TKP-TK20 group (**b**). Red fonts represent up-regulated DEGs. Green fonts represent down-regulated DEGs.

We used the signaling pathway data in the KEGG database to conduct a more detailed analysis of the screened DEGs related to cell vacuolation. Currently, only 11 of the 21 core genes are annotated into the KEGG signaling pathway. In the TK5-TK20 group, the up-regulated expression of PORCN is related to cell proliferation and migration. PORCN is produced in the endoplasmic reticulum and catalyzes Wnt lipidation. Lipidated Wnt binds to its receptor Frizzled to promote cell proliferation. Nlgn1, as a postsynaptic adhesion molecule, cooperates with Nrxns to participate in the regulation of synaptic excitation. Grik5 is a member of glutamate receptors, and its expression plays a key role in the regulation of the central nervous system and cell proliferation and differentiation (Figure 7a). In the TKP-TK20 group, ttk, BUB1, MAD2L1 and Cdc20 are involved in the synthesis of the mitotic checkpoint complex (MCC) after the correct connection of chromatin kinetochores (KTs) and microtubules (MTs), and the combination with the anaphase-promoting complex or cyclosome (APC/C) to ensure sister chromosomes segregate correctly. Similarly, the expression of ccnb1 and cdk1 is also involved in cell cycle regulation, centrosome duplication, and chromosome segregation. As one of the two important components of the nuclear lamina, LMNB1 plays a central role in the condensation of chromosome regions and the distribution of heterochromatin. HMMR is a receptor for hyaluronic acid (HA) and is highly expressed in tissue stress and repair processes. RAD51 controls DNA fidelity by forming nucleoprotein filaments on single-stranded DNA (ssDNA) (Figure 7b).

### 3.7. Validation of Select Gene Expression Changes by qPCR

**Figure 8:**
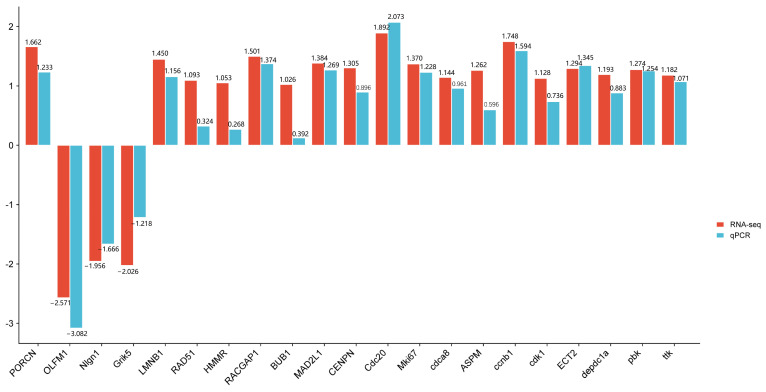
Comparison of RTPCR and RNASeq for 21 DEGs.

To verify the reliability of the transcriptome data, we performed qPCR verification on the 21 selected core genes (Figure 8). qPCR showed that the expression patterns of these DEGs were basically consistent with the results of RNA-seq analysis, verifying the accuracy and reliability of RNA-seq analysis.

## 4. Discussion

Cell culture technology plays a vital role in biomedical research and bioengineering. Through cell culture technology, researchers can culture and study living cells under controlled conditions to explore cell physiology, metabolism, signal transduction, and disease prevention and control mechanisms. In addition, cell culture technology is also widely used in fields such as drug screening, biopharmaceutical production, tissue engineering, and regenerative medicine [14]. Serum concentration is a crucial parameter in cell culture, which directly affects the cell growth, proliferation, and phenotype. Serum contains various components such as growth factors, hormones, and cell adhesion molecules, which can provide the nutrients and support needed by cells and promote their growth and proliferation [15]. Trypsin plays an important role in cell culture. It can effectively break down the extracellular matrix, reduce intercellular adhesion, and facilitate the dispersion and culture of single cells [16]. Our previous study found that increasing serum concentrations resulted in cell cavitation. Trypsin digestion improves this vacuolation as cells are passaged. Currently, no studies have been conducted to determine whether this vacuolization phenomenon is beneficial for the growth of fish cell lines. In this study, transcriptome analysis was used to explore DEGs related to cell vacuolation.

Cellular vacuolation is a common morphological phenomenon commonly observed in mammalian cells exposed to pathogens and various stimuli. Vacuolization usually accompanies cell death, but transient vacuolization can be observed during exposure to inducers and reversibly affects the cell cycle and migration, such as weakly basic amine-containing lipophilic compounds, the most known transient vacuolization [4]. All cells appear to retain the ability to form vacuoles, and vacuoles in cells are often viewed as an adaptive physiological response [6]. Henics et al. exposed mammalian cell lines to human serum ultrafiltrate to induce reversible cytoplasmic vacuolization. The results showed that the formation of vacuoles did not involve osmotic stress, and there was no obvious major metabolic damage in vacuolated cells; only intracellular water has restricted movement characteristics in vacuolated cells [5]. In the human body, transient cellular vacuolization can be seen in cardiomyocytes, where vacuolization gradually develops and then decreases with time [17]. Massive autophagic vacuolization may cause cellular stress and represents a frustrated attempt at adaptation [18].

GO analysis is a common method for understanding large-scale genomic data. On the one hand, GO functional analysis provides GO functional classification annotations of DEGs; on the other hand, it provides GO functional significance enrichment analysis of DEGs. KEGG analysis is a method used to grasp the regulation mechanisms within biological systems. It provides rich information about biological pathways, genomics, chemical substances et al. In the TK5-TK20 group and TKP-TK20 group, the significant enrichment GO term of ‘receptor binding’, ‘microtubule’, ‘polymeric cytoskeletal fiber’ et al. suggests that cell vacuolization is related to biological processes, such as the cell division cycle. The significant enrichment signaling pathway of the ‘Wnt signaling pathway’, ‘ECM-receptor interaction’ cell vacuolization is related to cell proliferation and other physiological processes.

In the TK5-TK20 group, we captured four core DEGs, of which only PORCN, Nlgn1, and Grik5 can be enriched in the KEGG signaling pathway. PORCN is a multichannel integral membrane enzyme expressed on the endoplasmic reticulum. PORCN palmitoylates Wnts and secretes them out of the cell membrane [19]. PORCN overexpression can change the position of β-catenin in a Wnt/β-catenin-dependent manner, and promote the transcription of downstream genes and invasion of HCC cells [20]. Wnts are lipid-modified signaling glycoproteins present in all metazoans that play key roles in development and homeostasis. PORCN is the only enzyme that catalyzes Wnt lipidation [21]. Wnt proteins are enzymatically lipidated by PORCN in the endoplasmic reticulum (ER) and bind to its transporter Wntless (WLS) as well as to its receptor Frizzled (FZD) for intracellular transport and secretion [22,23]. Excitatory synapses are formed and matured by the cooperative actions of synaptic organizers, such as neurexins (Nrxns), neuroligins (Nlgns) et al. [24]. Nlgn1 is a major component of the excitatory glutamatergic synaptic complex and plays a role in synaptic assembly and function [25]. As synaptogenesis marker, Nlgn1 can encode a transsynaptic protein and serve as a postsynaptic adhesion molecule involved in the regulation of glutamate transmission [26]. Grik5 is a member of glutamate receptors [27]. Grik5 is responsible for encoding KA2 (a kainate-preferring glutamate receptor subunit), predominantly found in the central nervous system where it functions as a ligand-gated ion channel. This gene has been identified as critical for the proliferation, differentiation, and engraftment capabilities of primary human leukemia cells during xenotransplantation studies [28]. Most of the current research on Grik5 is related to the proliferation and metastasis of cancer cells [29]. In this study, the increase in serum concentration led to the up-regulation of PORCN, a gene related to cell proliferation, and the down-regulation of Nlgn1 and Grik5, genes related to the nervous system. Based on the above information, we speculate that the phenomenon of cell vacuolization caused by excessive serum concentration occurs because it accelerates the rate of cell proliferation and migration.

In the TKP-TK20 group, we captured 21 core DEGs, of which only LMNB1, RAD51, HMMR, BUB1, Cdc20, MAD2L1, ccnb1, cdk1, and ttk. can be enriched in the KEGG signaling pathway. BUB1 encodes a serine/threonine protein kinase that is critical for the localization of the mitotic checkpoint protein BUBR1. ttk is another mitotic checkpoint protein associated with cell proliferation and responsible for the accurate segregation of chromosomes during mitosis [30]. The spindle assembly checkpoint protein BubR1 is thought to act by forming an inhibitory complex with Cdc20 [31]. The Bub1-Bub3 complex, the crucial regulator involved in the whole process of chromosome alignment and separation during mitosis, controls the fasting-induced lipid catabolism [32]. Loss of the BUB3-BUB1 complex results in telomere replication defects, including fragile and shortened telomeres [33]. Initial activation of the spindle assembly checkpoint (SAC) signaling occurs by recruiting the Bub3-Bub1/BubR1 (Bub) complex to the chromopetal kinetochore (KT), phosphorylating Bub1, and recruiting the Mad1-Mad2 complex together with Cdc20 to the unconnected site, and then Mad2 undergoes a conformational change [34]. After the KT and microtubules (MTs) are properly connected, the MCC is disassembled [35]. The Mad2-Cdc20 complex is released from the KT and combines with cytoplasmic Bub3-BubR1 to form the mitosis checkpoint complex (MCC) that binds the anaphase-promoting complex or cyclosome (APC/C), a crucial factor of the spindle assembly checkpoint (SAC), to ensure the bi-directional attachment and proper segregation of all sister chromosomes [36]. LMNB1 is ubiquitously expressed in all cell types and is one of the two important components of nuclear lamina [37]. Lamins assemble into nonpolar, bundled filaments that provide support against strain and that scaffold the genome [38]. LMNB1 expression is crucial for preserving the structural integrity of the nucleus and organizing chromatin [39]. Following the suppression of LMNB1, there was a notable decrease observed in the cell proliferation rate, wound healing capacity, transwell migration, and colony formation efficiency [40]. Lamins are essential constituents forming the structural framework of the nuclear lamina and LMNB1 plays a central role in chromosome region condensation, heterochromatin distribution, and the regulation of gene expression and splicing [37]. Hyaluronic acid (HA) is a prevalent glycosaminoglycan found abundantly in the extracellular matrix (ECM) [41]. HA fragments are generated by reactive oxygen/nitrogen species (ROS/RNS) and hyaluronidases produced during tissue stress and repair [42]. In a rat model of myocardial infarction, upregulation of HA and HMMR suggests that the HA pathway may play a key role in cardiac regenerative repair [43]. RAD51 and its gene family play crucial roles in maintaining the accuracy of DNA by regulating various processes such as repairing double-strand breaks, managing replication stress, and facilitating meiosis [44]. RAD51 is an ATPase that forms a nucleoprotein filament on single-stranded DNA (ssDNA), whereas binding to double-strand DNA (dsDNA) is inhibitory [45]. The CCNB1 and CDK1 genes encode the proteins of CyclinB1 and CDK1, respectively. These proteins collaborate to regulate the cell cycle, control centrosome duplication, and facilitate the proper segregation of chromosomes [46]. CDK1-CCNB1, also known as Cyclin B-dependent kinase, drives the initiation of mitosis and further prevents the premature exit from mitosis by activating the spindle checkpoint [47]. In Xenopus embryos, the cell cycle is driven by an autonomous biochemical oscillator, which consists of CDK1, Cdc25, Wee1, and Myt1. This oscillator controls the periodic activation and deactivation of cyclin B1-CDK1 [48]. The activation of Cyclin B1 and CDK1, along with their complex formation during the G2/M transition, is tightly regulated by various factors, such as CDC25 and Wee1 [49]. Recently, the death-effector domain (DED) containing protein, DEDD, was identified as a novel inhibitor of mitotic Cdk1/cyclin B1, influencing cell size [50]. Compared with the TKP group, the up-regulated genes in the TK20 group are basically related to mitosis, such as (BUB1, ttk, Mad2, Cdc20, CDK1, CCNB1). In addition, excessive serum concentration also led to the up-regulated expression of the nuclear stability-related genes LMNB1 and the tissue stress and repair-related genes HMMR.

## 5. Conclusions

In this study, transcriptome analysis results showed that excessive serum concentration could accelerate cell proliferation compared with cells cultured with low-concentration serum. Compared with vacuolated cells, the high expression of genes related to cell mitosis and cell homeostasis in cells with improved vacuolization after several passages indicates that there is a regulatory system related to adaptation and stress repair in the cells, which can maintain cell stability to a certain extent. However, persistent cellular vacuolization is often accompanied by cell death. Our results support the theory that transiently reversible cell vacuolization is a symbol of adapting to environmental changes, relieving survival pressure, and improving cell survival. Furthermore, the phenomenon of a large number of cell vacuolations can also serve as a warning, prompting us to adjust the culture conditions in time to avoid irreversible damage to the cells.

## Figures and Tables

**Table 1:** Sequencing information of nine cDNA libraries.

Sample	Raw Data	Clean Data	Q20 (%)	Q30 (%)	GC (%)	Total Mapped (%)
TK20-1	47,664,300	47,444,024	97.71%	93.36%	48.98%	75.22%
TK20-2	41,142,298	40,955,680	97.49%	92.83%	48.97%	74.54%
TK20-3	38,962,908	38,782,894	97.42%	92.80%	49.10%	74.36%
TK5-1	42,437,616	42,266,802	97.80%	93.52%	48.95%	72.10%
TK5-2	49,232,362	48,957,950	97.14%	92.13%	49.07%	71.62%
TK5-3	51,970,388	51,665,498	97.32%	92.62%	48.78%	71.43%
TKP-1	45,281,128	45,052,994	97.82%	93.63%	49.28%	79.38%
TKP-2	43,641,442	43,389,930	97.28%	92.47%	49.47%	79.22%
TKP-3	49,557,298	49,263,406	97.28%	92.46%	49.34%	79.34%

## Data Availability

The original contributions presented in the study are included in the article and Supplementary Material; further inquiries can be directed to the corresponding author.

## Supplementary Materials

The following supporting information can be downloaded at: https://www.mdpi.com/article/10.3390/biology13070545/s1, Table S1: Information of primers used in fluorescence quantitative PCR experiments. Table S2: The top 20 GO terms enriched in TK5-TK20 group and TK5-TKP group.. Figure S1: Vacuolization of Japanese flounder FG cell line caused by excessive serum concentration. (a) Cells cultured in 5% FBS medium for two weeks; (b) Cell vacuolation occurs after two weeks of culture in 20% FBS medium; (c) The vacuolization of cells improved after cells cultured in 20% FBS medium were digested and passaged with trypsin; (d) Cells cultured in 20% FBS medium were digested with trypsin and cultured until the cells returned to normal from the vacuolated state. Arrows indicate vacuolated cells. Scale bars: 100 µm.
