# Supplementary figures and images for: Transcriptome Analysis of Transiently Reversible Cell Vacuolization Caused by Excessive Serum Concentration in Scophthalmus maximus
Source: Biology (Basel). 2024 Jul 19;13(7):545. doi: 10.3390/biology13070545 (PMC11274238; doi:10.3390/biology13070545)

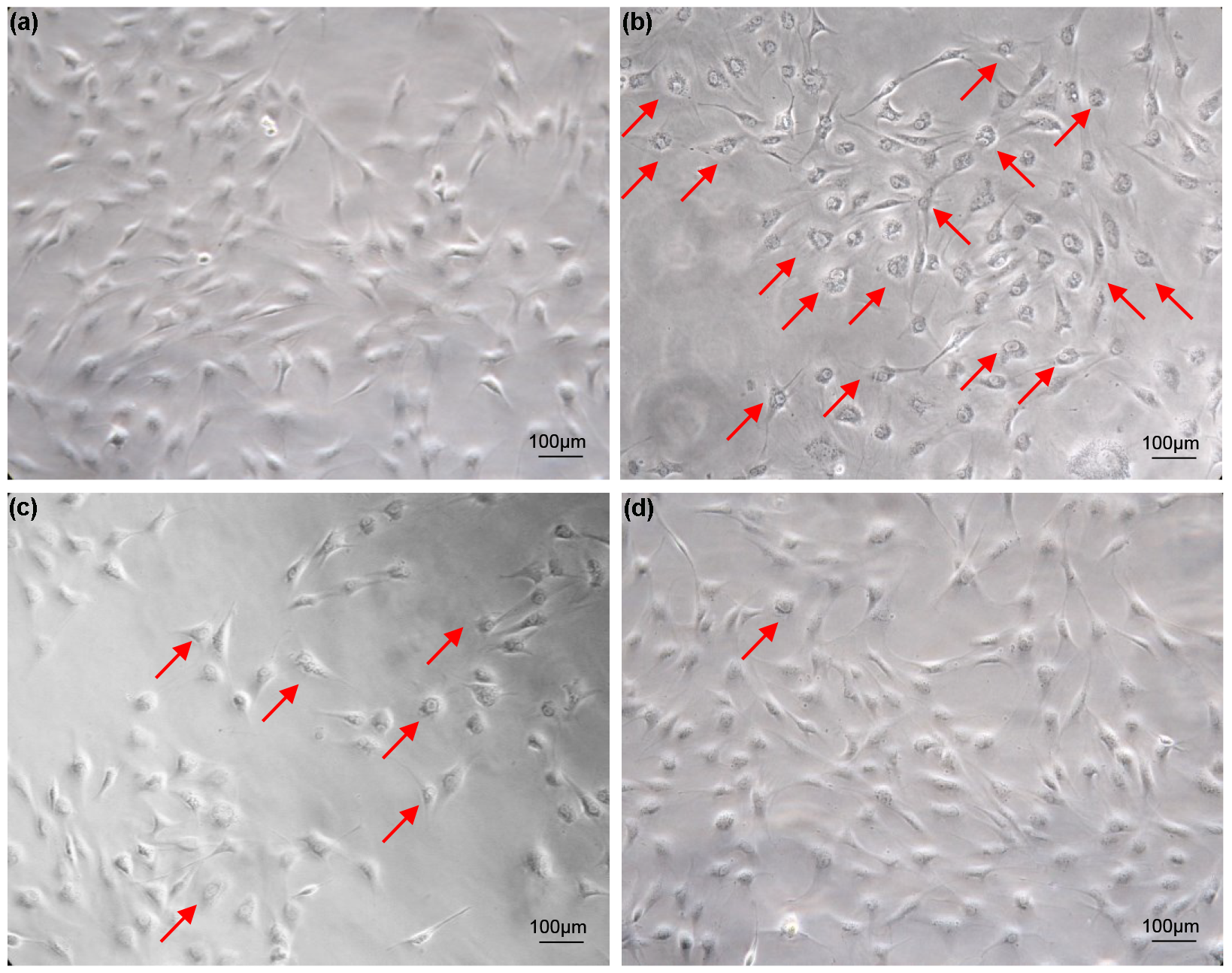

Supplement: Supplementary file 1 [file biology-13-00545-s001.zip › Figure1.tif]
